# Prevalence of metabolic syndrome in mainland china: a meta-analysis of published studies

**DOI:** 10.1186/s12889-016-2870-y

**Published:** 2016-04-01

**Authors:** Ri Li, Wenchen Li, Zhijun Lun, Huiping Zhang, Zhi Sun, Joseph Sam Kanu, Shuang Qiu, Yi Cheng, Yawen Liu

**Affiliations:** Department of Epidemiology and Biostatistics, School of Public Health, Jilin University, Changchun City, Jilin Province China; Department of Neurotrauma, First Hospital of Jilin University, Changchun City, Jilin Province China; Department of Library, First Hospital of Jilin University, Changchun City, Jilin Province China; Department of Psychiatry, VA Medical Center, Yale University School of Medicine, West Haven, USA; Clinical Laboratory of China-Japan Union Hospital of Jilin University, Changchun City, Jilin Province China; Department of Cardiovascular Center, First Hospital of Jilin University, Changchun City, Jilin Province China

**Keywords:** Prevalence, Metabolic syndrome X, Meta-analysis

## Abstract

**Background:**

Metabolic syndrome (MS) comprises a set of conditions that are risk factors for cardiovascular diseases and diabetes. Numerous epidemiological studies on MS have been conducted, but there has not been a systematic analysis of the prevalence of MS in the Chinese population. Therefore, the aim of this study was to estimate the pooled prevalence of MS among subjects in Mainland China.

**Methods:**

We performed a systematic review by searching both English and Chinese literature databases. Random or fixed effects models were used to summarize the prevalence of MS according to statistical tests for heterogeneity. Subgroup, sensitivity, and meta-regression analyses were performed to address heterogeneity. Publication bias was evaluated using Egger’s test.

**Results:**

Thirty-five papers were included in the meta-analysis, with a total population of 226,653 Chinese subjects. Among subjects aged 15 years and older, the pooled prevalence was 24.5 % (95 % CI: 22.0–26.9 %). By sex, the prevalences were 19.2 % (95 % CI: 16.9–21.6 %) in males and 27.0 % (95 % CI: 23.5–30.5 %) in females. The pooled prevalence of MS increased with age (15–39 years: 13.9 %; 40–59 years: 26.4 %; and ≥60 years: 32.4 %). Individuals living in urban areas (24.9 %, 95 % CI: 18.5–31.3 %) were more likely to suffer from MS than those living in rural areas (19.2 %, 95 % CI: 14.8–23.7 %). Hypertension was the most prevalent component of MS in males (52.8 %), while the most prevalent component of MS for females was central obesity (46.1 %).

**Conclusions:**

Our systematic review suggested a high prevalence of MS among subjects in Mainland China, indicating that MS is a serious public health problem. Therefore, more attention should be paid to the prevention and control of MS.

**Electronic supplementary material:**

The online version of this article (doi:10.1186/s12889-016-2870-y) contains supplementary material, which is available to authorized users.

## Background

Metabolic syndrome (MS) is characterized by a cluster of metabolic disorders, such as high blood pressure, hyperglycaemia, central adiposity, and dyslipidemia [1, 2]. MS is considered to be risk factor for coronary heart disease, other cardiovascular diseases (CVD), stroke, and type 2 diabetes mellitus [3, 4]. The prevalence of MS is increasing in both developed and developing countries and has become a serious public health problem worldwide [5–8].

China is the world’s largest developing country and is experiencing an epidemic of MS [9]. The Nantong MS Study conducted between 2007 and 2008 in south China showed that the prevalence of MS was 15.2 % [10]. A study in north China revealed that the prevalence of MS was 21.6 % in males and 34.3 % in females [11]. The prevalence may vary due to the diverse populations of different regions, cultural behaviours, lifestyle habits, and the use of different diagnosis criteria [2, 9, 12]. Although a number of epidemiological studies on MS were conducted in the Chinese population in recent years, very little nationwide information exists on the prevalence of MS. A nationwide estimate of MS prevalence in the China population would contribute to the planning and implementation of relevant public health strategies. Therefore, we performed a systematic review of epidemiological studies of MS to estimate the prevalence of MS among subjects in Mainland China.

## Methods

### Search strategy

We searched for epidemiological studies on MS from the several electronic databases, including Medline, Embase, the China National Knowledge Infrastructure (CNKI), and the Wanfang and Chongqing VIP. The following search strategy was used: (‘Metabolic syndrome’ OR ‘MS’ OR ‘MetSyn’) AND (‘prevalence’ OR ‘epidemi*’) AND (‘Chinese’ OR ‘China’ OR ‘Mainland’). All studies published from January 1, 2005 to April 30, 2015 were searched. Unpublished studies were not retrieved. The search language was limited to English and Chinese.

### Inclusion and exclusion criteria

To satisfy the analysis requirements and reduce selection deviation, the selected studies were required to meet the following criteria: 1) a population-based study conducted in Mainland China; 2) a cross-sectional study or data; 3) sufficient information of sample size and crude prevalence of MS; 4) a sample size > 500; 5) participants aged 15 years and older; and 6) the use MS diagnostic criteria proposed by the International Diabetes Federation (IDF) in 2005 [13]. According to the IDF criteria, the participants were classified as having MS if they had central obesity (waist circumference ≥ 90 cm for men and ≥ 80 cm for females) plus any two of the following four abnormalities: a) Hypertension: systolic blood pressure ≥ 130 mmHg, diastolic blood pressure ≥ 85 mmHg, or treatment of previously diagnosed hypertension; b) Hypertriglyceridemia: ≥ 1.7 mmol/L triglycerides or specific medical treatment for lipid abnormalities; c) Hypo-HDL-cholesterol: < 1.03 mmol/L HDL cholesterol for men or < 1.29 mmol/L for females; and d) Raised fasting glucose: overnight ≥ 5.6 mmol/L plasma glucose or previously diagnosed diabetes. We excluded studies that investigated specific occupations, volunteers, and hospital-based populations. If there were multiple articles based on the same population, only the study that reported the most detailed data was included.

### Data extraction and quality assessment

All searched articles from different electronic databases were combined in Endnote, and duplicates were removed. Two researchers independently screened the titles and abstracts and reviewed the full text of the eligible citations. If they were in disagreement, a third reviewer made the final decision. For each included study, two researchers independently extracted the following information: general information (e.g., first author, title, journal, and publication year); study characteristics (including study period, study area, study design, sample source, sample selection method, diagnostic criteria, and sample size); and all possible participant information(e.g., sex ratio, age, prevalence of MS, age-specific prevalence of MS, the prevalence of central obesity, hypertension, raised fasting glucose, hypertriglyceridaemia, and low high-density liprotein (HDL) cholesterol). Two researchers independently assessed the quality of each included study using observational study criteria that were recommended by the Agency of Healthcare Research and Quality [14]. Only when two reviewers agreed was the study included in the meta-analysis. The retained articles were required to have a quality score of at least 6 of 11.

### Statistical analysis

We used a systematic analysis approach to calculate the pooled prevalence of MS from all eligible studies. A random or fixed effects model was selected to summarize the prevalence of MS, using statistical tests for heterogeneity. Heterogeneity among studies was assessed using Cochran’s Q test and I^2^ statistic, which shows the percentage of variation across studies (with values of 25, 50, and 75 % indicating low, moderate, and high degrees of heterogeneity, respectively) [15, 16]. If the data showed low or moderate heterogeneity (I^2^ < 50 %), a fixed-effect model was used; otherwise, a random-effect model was used. Subgroup analyses by geographic region, age, sex, and the year of screening were performed to address heterogeneity. Additionally, a meta-regression was conducted to explore potential sources of heterogeneity. Variables such as the year of publication, year of screening, response rate, geographic area (e.g., northern *vs*. southern China), sex ratio (males vs. females), sample size, age range, and quality score were used to perform the meta-regression. Additionally, sensitivity analysis (i.e., recalculating the pooled estimate by omitting studies with low scores) was performed to assess the influence of any particular study on the pooled estimate.

Publication bias was evaluated using Egger’s Test, and independent t-tests were performed as appropriate. The significance level was set at a *P* value of less than 0.05. All statistical analyses were performed using Stata version 12.0 (College Station, Texas) and SPSS version 20.0 (SPSS Inc., Chicago, USA).

## Results

### Search results and included subjects

**Fig. 1:**
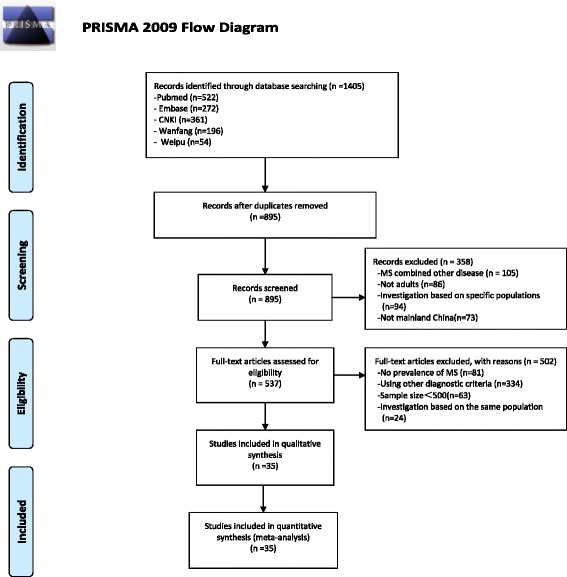
Flow diagram of studies included in the systematic review

A total of 1405 citations were searched. Of these, 510 duplicates were removed, and 358 citations were excluded after reading the titles and abstracts. Five hundred thirty-seven articles were further excluded after reviewing the full texts. In total, 35 eligible studies were included in the meta-analysis, which involved a total of 226,653 subjects. The flow diagram of the search process is shown in Fig. 1. Among the 35 published papers, 22 were written in Chinese and 13 were written in English. All of the included studies were cross-sectional surveys. Thirty-one studies reported data on males (*n* = 94,241) and 32 studies reported data on females (*n* = 127,079). Sixteen and 17 studies were conducted on the populations of south and north China, respectively, and two nationwide studies were conducted. Table 1 shows the detailed characteristics of the 35 studies selected. On a quality assessment scale, seven studies scored 6, and 28 articles scored between 6 and 10. Additional file 1 shows the score of each study.

**Table 1:** Characteristic of studies on the prevalence of metabolic syndrome

NO.	First author	Publication year	Screening year	Region	Area	Age range	Sex	Case	Sample	Prevalence (%)	Score
							(M/F)	(n)	size		
1	Zhang YH et al. ^a^[37]	2014	2008	Beijing	Northern	≥18y	0.94	161	724	22.2	7
	Zhang YH et al. ^b^[37]	2014	2011	Beijing	Northern	≥18y	0.67	279	864	32.3	7
2	Cao YL et al. [38]	2015	2013	Hunan	Southern	≥18y	1.16	826	3108	26.58	7
3	Chen QY et al. [39]	2007	2003–2005	Guangxi	Southern	≥15y	1.28	3582	27,240	13.15	6
4	Li H et al. [40]	2013	2011	Guizhou	Southern	40–79y	0.37	4063	10,016	40.57	7
5	Fu SY et al. [41]	2010	2007	Heilongjiang	Northern	35–91y	0.802	1472	5984	24.6	8
6	Tao R et al. [42]	2015	2010	Jiangsu	Southern	18–95y	0.901	2472	8380	29.5	8
7	Xu DM et al. [43]	2010	2007–2008	Henan	Northern	18–88y	0	89	579	15.3	7
8	Lu W et al. [44]	2006	2002–2003	Shanghai	Southern	15–74y	0.745	2509	14,327	17.51	10
9	Hu Y et al. [45]	2008	2007	Liaoning	Northern	65–94y	1.96	633	2730	23.19	7
10	Wang WC et al. [46]	2014	2011	Hebei	Northern	≥45y	0.543	307	1447	21.2	8
11	Du YH et al. [47]	2007	2005–2006	Shanxi	Northern	20–93y	0.488	979	3869	25.3	9
12	Yu H et al. [48]	2012	2010	Tianjin	Northern	30–60y	1.02	546	2993	18.24	8
13	Yu L et al. [49]	2008	2003–2004	Neimonggu	Northern	≥20y	0.693	530	2536	20.9	8
14	Zhang SQ et al. [50]	2007	2002	Zhejiang	Southern	≥50y	0.672	288	1187	24.26	6
15	Zhao FC et al. [51]	2009	2007	Xinjiang	Northern	20–74y	0.69	823	3293	24.99	7
16	Li SJ et al. [52]	2012	2010	Zhejiang	Southern	≥18y	0.775	179	600	29.83	7
17	Deng M et al. [53]	2014	2011–2012	Chongqing	Southern	≥35y	0.713	1092	5384	20.28	6
18	Ye QY et al. [54]	2012	2010	Zhejiang	Southern	≥18y	0.88	339	1248	27.16	7
19	Ta JGL et al. [55]	2013	2010	Xinjiang	Northern	≥20y	0.457	817	2138	38.2	7
20	Li CH et al. [56]	2012	2010	Xinjiang	Northern	≥18y	0.97	730	3442	21.2	8
21	Li YQ et al. [57]	2014	2012	Guangdong	Southern	18–75y	0.595	383	1724	22.22	6
22	Zhao Y et al. [33]	2010	2008–2009	Ningxia	Northern	≥25y	null	355	1612	22	8
23	Sun M et al. [58]	2014	2011	Jiangsu	Southern	≥40y	0.6	2973	7489	39.7	9
24	Lao XQ et al. [9]	2014	2010	Guangdong	Southern	≥20y	0.82	872	3561	24.5	8
25	Yu M et al. [59]	2014	2009	Zhejiang	Southern	19–79y	1	1242	8169	15.2	6
26	He Yao et al. [60]	2006	2001–2002	Beijing	Northern	60–95y	0.67	1081	2334	46.3	8
27	Zhou HC et al. [61]	2014	2007–2008	14 provinces	National	≥20y	0.66	11,244	45,157	24.9	8
28	Xi B et al. [34]	2013	2009	9 provinces	National	≥18y	0.871	1767	7488	23.6	7
29	Peng X et al. [62]	2009	2007	Hunan	Southern	≥18y	0.99	260	1709	15.2	7
30	Cai H et al. [2]	2012	2007–2008	Jiangsu	Southern	18–74y	0	2965	13,505	22	6
31	Zhao J et al. [11]	2011	2006	Shandong	Northern	35–74y	0.688	1082	5355	20.2	7
32	Tan XU et al. [63]	2009	2002–2003	Neimonggu	Northern	≥20y	0.693	530	2536	20.9	7
33	Li G et al. [64]	2010	2005	Beijing	Northern	≥18y	0.652	4587	16,442	27.9	6
34	Zhao YL et al. [65]	2014	2010	Shanxi	Northern	18–80y	0.529	407	2990	13.6	8
35	Xu F et al. [66]	2011	2009–2010	Jiangsu	Southern	18–74y	0.878	1213	4493	27	7

Study[37] has two parts; ^a^the screening year of one part is 2008, ^b^the screening year of the other part is 2011

### Prevalence of metabolic syndrome

**Fig. 2:**
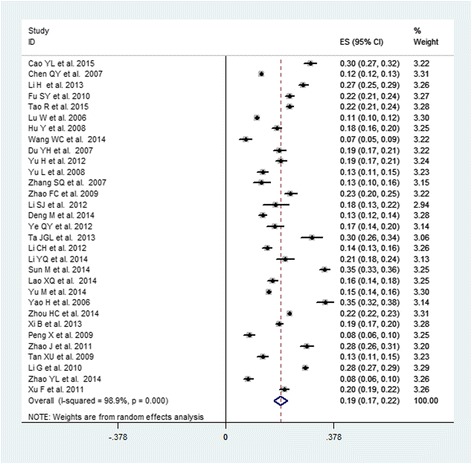
Forest plot of the studies of males

**Fig. 3:**
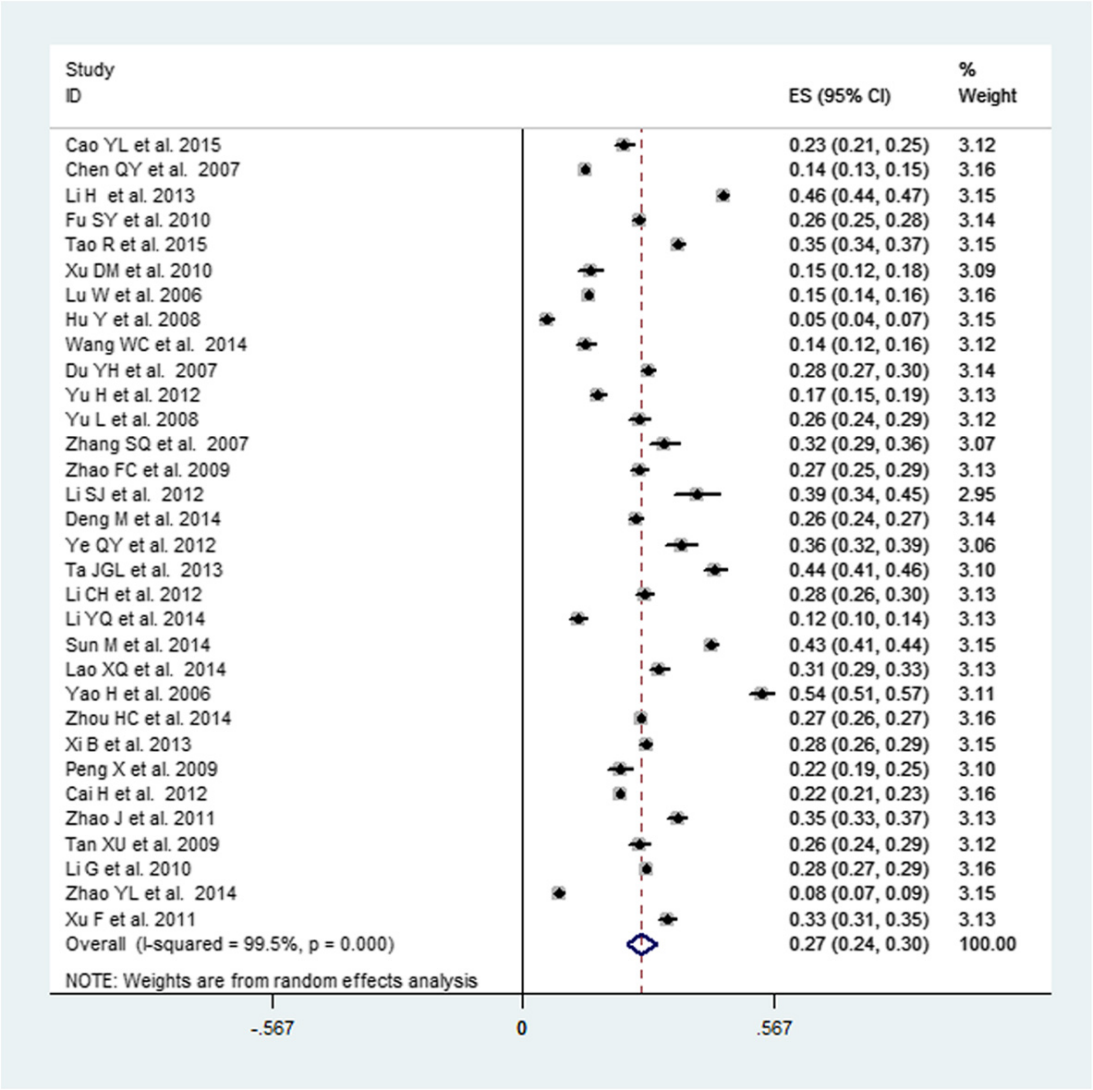
Forest plot of the studies of females

The pooled prevalence of MS among Chinese subjects was 24.5 % (95 % CI: 22.0–26.9 %), with a high-level between-study heterogeneity (*I*^2^ = 99.5 %, *P* < 0.0001). Table 2 demonstrates the pooled prevalence of all subgroups stratified by sex, geographic area, study period, and age range. The pooled prevalence in males (19.2 %, 95 % CI: 16.9–21.6 %, Fig. 2) was lower than that of females (27.0 %, 95 % CI: 23.5–30.5 %, Fig. 3). The t-tests showed that the prevalence of MS was significantly different between males and females (*P* = 0.002). The prevalences of MS in the populations of north and south China were similar (24.4 and 24.6 %, respectively). The pooled prevalence of MS in the population living in rural areas (19.2 %, 95 % CI: 14.8–23.7 %) was lower than was observed in urban areas (24.9 %, 95 % CI: 18.5–31.3 %). The pooled prevalence of MS increased with time. The pooled prevalence was 23.8 % (95 % CI: 17.7–29.9 %) during 2000–2005, increasing to 22.3 % (95 % CI: 20.3–24.3 %) during 2005–2010 and 27.0 % (95 % CI: 22.2–31.8 %) during 2010–2015. Additionally, the summarized prevalence of MS increased with age. The pooled prevalences of MS for specific age ranges were 13.9 % (95 % CI: 9.5–18.2 %) for subjects aged 15–39 years, 26.4 % (95 % CI: 20.5–32.3 %) for subjects aged 40–59 years, and 32.4 % (95 % CI: 26.1–38.8 %) for subjects aged ≥ 60 years. The prevalence of MS increased with age in males, peaking in the 40–59 year age group and decreasing thereafter. The prevalence of MS also increased with age in females, peaking in the ≥ 60 years group.

**Table 2:** Prevalence of MS according to a different category

Category	Subgroup	NO.of study	Prevalence (95 % CI)(%)	Sample	*I* ^*2*^ *(%)*	*P*	*P*(Egger’s Test)
Total		36	24.5(22.0–26.9)	226,653	99.5	<0.001	0.072
Geographic region	Northern	17	24.4(21.4–27.3)	61,868	98.7	<0.001	0.976
	Southern	16	24.6(20.2–29.1)	112,140	99.7	<0.001	0.036
	Urban	7	24.9(18.5–31.3)	24,560	99.3	<0.001	0.060
	Rural	16	19.2(14.8–23.7)	53,268	99.5	<0.001	0.048
Sex	Male	31	19.2(16.9–21.6)	94,241	98.9	<0.001	0.150
	Female	32	27.0(23.5–30.5)	127,079	99.8	<0.001	0.141
Screening year	2000–2005	6	23.8(17.7–29.9)	50,160	99.6	<0.001	0.051
	2005–2010	15	22.3(20.3–24.3)	121,109	98.4	<0.001	0.322
	2010–2015	15	27.0(22.2–31.8)	55,384	99.4	<0.001	0.571
Age-specific group(y)	15–39	10	13.9(9.5–18.2)	20,273	98.8	<0.001	0.017
	40–59	12	26.4(20.5–32.3)	38,484	99.4	<0.001	0.258
	≥60	12	32.4(26.1–38.8)	18,652	98.8	<0.001	0.955
Male	15–39	5	14.9(6.8–23.0)	8585	99.0	<0.001	0.100
	40–59	7	23.4(16.3–30.5)	14,845	98.8	<0.001	0.279
	≥60	7	23.0(18.0–28.0)	7850	96.2	<0.001	0.292
Female	15–39	5	9.5(5.3–13.7)	9536	98.2	<0.001	0.069
	40–59	7	27.2(19.3–35.2)	19,586	99.3	<0.001	0.550
	≥60	7	42.9(34.5–51.3)	8800	98.4	<0.001	0.273

### Prevalence of components of metabolic syndrome

In terms of the different components of MS, the pooled prevalence estimates of central obesity, hypertension, high fasting plasma glucose, hypertriglyceridaemia, and low HDL cholesterol in males were 33.4, 52.8, 31.5, 32.9, and 27.4 %, respectively. For females, these estimates were 46.1, 40.1, 26.3, 27.7, and 40.4 %, respectively. The prevalence of hypertension in males was significantly higher than in females (*P* = 0.049). Table 3 shows the pooled prevalences of different the components of MS.

**Table 3:** Prevalence of different components of MS

Types	Sex	NO. of study	Sample	Pooled prevalence(95 % CI)(%)	Median(%)	Minimum(%)	Maximum(%)	*t*	*P*
Central obesity	Male	14	38,434	33.4(25.3–41.5)	26	18	68.8	−2.034	0.052
	Female	15	44,646	46.1(37.0–55.2)	47.2	6.8	77.5		
Hypertension	Male	14	38,434	52.8(45.3–60.4)	30.3	52	79	2.066	0.049
	Female	15	44,646	40.1(32.2–48.0)	41.3	15	75.9		
High Fasting Plasma Glucose	Male	14	38,434	31.5(25.3–37.8)	31	10.2	52.3	0.981	0.335
	Female	15	44,646	26.3(19.0–33.6)	24	3.4	52.5		
Hypertriglyceridaemia (TG)	Male	14	38,434	32.9(27.5–38.3)	32.9	11.6	53.8	1.189	0.245
	Female	15	44,646	27.7(22.0–33.4)	27.1	7.3	56.3		
Low HDL-C	Male	14	38,434	27.4(22.2–32.5)	27.2	5	55.5	−1.991	0.057
	Female	15	44,646	40.4(30.6–50.2)	36.3	1.4	70.4		

### Sensitivity analysis and meta-regression

Seven citations had a quality score of 6, the lowest among the included studies. In the sensitivity analysis, we noticed a slight change in the pooled MS prevalence estimate (from 24.5 to 25.4 %) when we omitted these seven studies. Egger’s linear regression test (*P* = 0.072) suggested no significant publication bias.

A high level of heterogeneity between studies and subgroups was observed (*P* < 0.001, *I*^*2*^ *=* 96.2–99.8 %). We performed a meta-regression to take this heterogeneity into account. In the univariate meta-regression and multivariable analyses, only the variable of age was significantly associated with heterogeneity (*P* = 0.01, *P* = 0.02, respectively) (Table 4).

**Table 4:** Results of meta-regression for the prevalence of metabolic syndrome

Covariate	Meta-regression coefficient	95 % confidence interval	*P* value	Variance explained (%)
Univariate analyses				
Sex ratio(male *vs.* female)	0.846	0.6195–1.155	0.283	1.83
Area(northern *vs.* southern)	1.0104	0.8486–1.2031	0.904	−3.14
Quality score	1.0044	0.9370–1.1641	0.421	−0.96
Year of screening	1.1046	0.9617–1.2687	0.153	3.62
Sample size, continuous	1.0000	0.9999–1.0001	0.519	−1.65
Age group(15 ~ =1, 40 ~ =2, 60 ~ =3)	1.2625	1.0615–1.5017	0.010	17.63
Year of publication	1.0988	0.8671–1.3924	0.424	−0.90
Multivariable analyses				26.74
Sex ratio(male *vs.* female)	−0.2331	−0.5318–0.6585	0.121	
Area(northern *vs.* southern)	0.0095	−0.1543–0.1734	0.906	
Quality score	0.0285	−0.0746–0.1315	0.576	
Year of screening	0.0906	−0.9722–0.2785	0.331	
Sample size, continuous	3.28e^’-7^	−0.0000–0.0001	0.952	
Age group(15 ~ =1, 40 ~ =2, 60 ~ =3)	0.3035	0.1187–0.4884	0.002	
Year of publication	0.9168	−0.2391–0.4225	0.574	

## Discussion

Our systematic review of observational studies conducted in the last decade included 35 studies that involved a total of 226,653 participants in Mainland China and covered most regions of the country. The definitions of IDF and the US National Cholesterol Education Program Adult Treatment Panel III (NCEP ATP III) are widely used in China [9]. The IDF criteria recognize and emphasize differences in waist circumference for Chinese populations [17]. Thus, the IDF criteria were adopted in our meta-analysis.

Our meta-analysis revealed that the pooled estimate of MS prevalence among subjects in Mainland China was 24.5 %. This estimate was higher than the prevalence of 16.5 % observed in China in 2000 and approached the worldwide prevalence of 20–25 % [5, 18]. The prevalence of MS has recently increased in developing countries. Several studies have reported a high prevalence of MS in Malaysia (27.5 %), India (28.2 %), Philippines (19.7 %), Nigeria (28.1 %), Brazil (29.6 %), Turkey (44.0 %), and Iran (36.9 %) [19–25]. As the largest developing country, China is experiencing an emerging epidemic of MS, which might be related to rapid economic development and urbanization [9]. Rapid industrialization and urbanization can lead to accelerating changes in lifestyle and nutrition. The prevalences of obesity and overweight have increased dramatically in China due to the changes in the lifestyle of the population, and some of these changes are independent factors that contribute to MS. Data from the China Health and Nutrition Survey shows that the age-adjusted prevalence of obesity increased from 3.75 % in 1991 to 11.3 % in 2011, and the prevalence of overweight was up to 42.3 % in 2011 [26]. In addition, another major factor driving MS growth is likely the ageing of the Chinese population. Studies have shown an increased prevalence of MS with age [19, 27]. Data from the National Bureau of Statistics in 2011 showed that people aged 60 and older accounted for 13.26 % of the Chinese population, with those 65 years and older representing 8.87 % of the population. These data show that China is now an ageing society [28].

Our systematic review showed that MS was more common in females than in males (27.0 vs. 19.2 %), a result that is in line with previous findings [19, 27, 29]. Menopause may have effects on the high prevalence of MS among females. Post-menopausal status is associated with an increased risk of central obesity and insulin resistance [30]. Our meta-analysis discovered that central obesity was the most prevalent component of MS in females. Moreover, a relationship was observed between the prevalence of MS and age in both males and females, which is consistent with other studies [31, 32]. The increased prevalence of MS with age can be attributed to similar age-related trends in all components of MS [9, 33]. Additionally, individuals living in urban areas were more likely to suffer from MS than those living in rural areas. Unhealthy lifestyles in urban area, including decreased physical activity, excessive intake of animal fat and salt, and low intake of fruits and vegetables might explain the difference in MS prevalence between the two regions [34].

There is an emerging MS epidemic in Mainland China, and it has become a serious public health problem. MS increases the risk for morbidity and mortality of cardiovascular disease and is associated with an increased risk of diabetes [5]. Studies have shown that the components of the syndrome tend to aggregate in individuals, and this clustering effect is associated with a worse prognosis than exhibiting a single component [35, 36]. Our results showed that MS was highly prevalent, especially in female, elderly participants and those living in urban areas. These data may be useful for the Chinese government in its formulation of guidelines to prevent, screen for and treat MS.

### Strengths and limitations

The overall quality of the studies included in our systematic review was good; therefore, the sensitivity analysis did not show major differences in the meta-analysis results when studies with the lowest quality scores were omitted. Our meta-analysis included 35 published studies with a large sample size. Nevertheless, our study had some limitations. First, we used the IDF criteria as our diagnosis criteria, and studies based on other diagnosis criteria were not included in our meta-analysis. Second, although most of the included studies had a large sample size that could generate an accurate estimation, the overall analysis revealed a high heterogeneity. Additionally, meta-regression and subgroup analyses did not indicate enough factors to explain the observed heterogeneity. We propose that other factors, such as cigarette smoking, alcohol consumption, stress, and physical inactivity may influence MS heterogeneity. Because of the limited information on these aspects, we could not perform further analyses. Third, the distribution of healthcare resources in Mainland China is unbalanced, with more economically developed areas having better access to health care facilities. This factor may have contributed to more diagnoses and, therefore, a higher reported prevalence in certain studies of different regions in Mainland China.

## Conclusion

To the best of our knowledge, this was the first systematic review to estimate the pooled prevalence of MS among subjects in Mainland China. Our systematic review indicates a high prevalence of MS among subjects in Mainland China. Information on how MS and its components are distributed could provide a great deal of insight into MS and assist in the planning and implementation of future prevention and control programmes.

## Additional file

Additional file 1: Table S1. Quality assessment scores of the included studies. (XLS 37 kb)

## References

[1] Isomaa B, Almgren P, Tuomi T, Forsén B, Lahti K, Nissén M (2001). Cardiovascularmorbidity and mortality associated with the metabolic syndrome. Diabetes Care.

[2] Cai H, Huang J, Xu G, Yang Z, Liu M, Mi Y (2012). Prevalence and determinantsof metabolic syndrome among women in Chinese rural areas. PLoS One.

[3] Martínez-Hernández A, Córdova EJ, Rosillo-Salazar O, García-Ortíz H, Contreras-Cubas C, Islas-Andrade S (2015). Association of HMOX1 and NQO1 Polymorphisms with Metabolic Syndrome Components. PLoS One.

[4] Rochlani Y, Pothineni NV, Mehta JL (2015). metabolic syndrome: does it differ between women and men?. Cardiovasc Drugs Ther.

[5] VidigalFde C, Ribeiro AQ, Babio N, Salas-Salvadó J, Bressan J (2015). Prevalence of metabolic syndrome and pre-metabolic syndrome in health professionals: LATINMETS Brazil study. DiabetolMetabSyndr.

[6] Scuteri A, Laurent S, Cucca F, Cockcroft J, Cunha PG, Mañas LR (2015). Metabolic syndrome across Europe: different clusters of risk factors. Eur J PrevCardiol.

[7] Beltrán-Sánchez H, Harhay MO, Harhay MM, McElligott S (2013). Prevalence and trends of metabolic syndrome in the adult US population, 1999–2010. J Am CollCardiol.

[8] Woo HD, Shin A, Kim J (2014). Dietary patterns of Korean adults and the prevalence of metabolic syndrome: a cross-sectional study. PLoS One.

[9] Lao XQ, Ma WJ, Sobko T, Zhang YH, Xu YJ, Xu XJ (2014). Dramatic escalation in metabolic syndrome and cardiovascular risk in a Chinese population experiencing rapid economic development. BMC Public Health.

[10] Jing X, Jian-Ping H, Guang-Fei X, De-Xi C, Gui-Yun W, Min Z, et al. Association of alcohol consumption and components of metabolic syndrome among people in rural China. NutrMetab. 2015;12. 10.1186/s12986-015-0007-4 PMC4350876 25745507

[11] Zhao J, Pang ZC, Zhang L, Gao WG, Wang SJ, Feng N (2011). Prevalence of metabolic syndrome in rural and urban Chinese population in Qingdao. J Endocrinol Invest.

[12] Zuo H, Shi Z, Hu X, Wu M, Guo Z, Hussain A (2009). Prevalence of metabolic syndrome and factors associated with its components in Chinese adults. Metabolism.

[13] Alberti KG, Zimmet P, Shaw J (2005). The metabolic syndrome-a new worldwide definition. Lancet.

[14] Rostom A, Dube’ C, Cranney A, Saloojee N, Richmond Sy, Mack D, et al. Celiac Disease. Rockville (MD): Agency for Healthcare Research and Quality (US); (Evidence Reports/Technology Assessments, No. 104.) Appendix D. Quality Assessment Forms. Available: http://www.ncbi.nlm.nih.Gov/books/NBK35156/Accessed 5 March 2014.

[15] Huedo-Medina TB, Sanchez-Meca J, Marin-Martinez F, Botella J (2006). Assessing heterogeneity in meta-analysis: Q statistic or I2 index?. Psychol Methods.

[16] Higgins JP, Thompson SG, Deeks JJ, Altman DG (2003). BMJ.

[17] Zimmet P, Magliano D, Matsuzawa Y, Alberti G, Shaw J (2005). The metabolic syndrome: a global public health problem and a new definition. J AtherosclerThromb.

[18] Yang W, Reynolds K, Gu D, Chen J, He J (2007). A comparison of two proposed definitions for metabolic syndrome in the Chinese adult population. Am J Med Sci.

[19] Rampal S, Mahadeva S, Guallar E, Bulgiba A, Mohamed R, Rahmat R (2012). Ethnic differences in the prevalence of metabolic syndrome: results from a multi-ethnic population-based survey in Malaysia. PLoS One.

[20] Nag T, Ghosh A (2015). Prevalence of metabolic syndrome in rural elderly of Asian Indian origin. Am J Hum Biol.

[21] SyRG LEJ, Reganit PF, Castillo-Carandang N, Punzalan FE, Sison OT (2014). Socio-demographic factors and the prevalence of metabolic syndrome among filipinos from the LIFECARE cohort. J AtherosclerThromb.

[22] OguomaVM NEU, Richards RS (2015). Prevalence of cardio-metabolic syndrome in Nigeria: a systematic review. Public Health.

[23] de Carvalho VF, Bressan J, Babio N, Salas-Salvadó J (2013). Prevalence of metabolic syndrome in Brazilian adults: a systematic review. BMC Public Health.

[24] Gundogan K, Bayram F, Gedik V, Kaya A, Karaman A, Demir O (2013). Metabolic syndrome prevalence according to ATP III and IDF criteria and related factors in Turkish adults. Arch Med Sci.

[25] Amirkalali B, Fakhrzadeh H, Sharifi F, Kelishadi R, Zamani F, Asayesh H (2015). Prevalence of Metabolic Syndrome and Its Components in the Iranian Adult Population: A Systematic Review and Meta-Analysis. Iran Red Crescent Med J.

[26] Mi YJ, Zhang B, Wang HJ, Yan J, Han W, Zhao J (2015). Prevalence and secular trends in obesity among Chinese adults, 1991–2011. Am J Prev Med.

[27] Kuk JL, Ardern CI (2010). Age and sex differences in the clustering of metabolic syndrome factors: association with mortality risk. Diabetes Care.

[28] Yu Y, Yang LJ, Yang TB (2013). China's aging population and the need for public health services. Chin J Gerontol.

[29] Pan JJ, Qu HQ, Rentfro A, McCormick JB, Fisher-Hoch SP, Fallon MB (2011). Prevalence of metabolic syndrome and risks of abnormal serum alanine aminotransferase in Hispanics: a population-based study. PLoS One.

[30] Fujimoto WY, Bergstrom RW, Boyko EJ, Chen K, Kahn SE, Leonetti DL (2000). Type 2 diabetes and the metabolic syndrome in Japanese Americans. Diabetes Res ClinPract.

[31] Park YH, Shin JA, Han K, Yim HW, Lee WC, Park YM (2014). Gender difference in the association of metabolic syndrome and its components with age-related cataract: the Korea National Health and Nutrition Examination Survey 2008–2010. PLoS One.

[32] Escobedo J, Schargrodsky H, Champagne B, Silva H, Boissonnet CP, Vinueza R (2009). Prevalence of the metabolic syndrome in Latin America and its association with sub-clinical carotid atherosclerosis: the CARMELA cross sectional study. Cardiovasc Diabetol.

[33] Zhao Y, Jin J, Liu XY, Xu HX, Yang JJ, Zhang YH (2010). Prevalence of the metabolic syndrome among rural original adults in NingXia, China. BMC Public Health.

[34] Xi B, He D, Hu YH, Zhou DH (2013). Prevalence of metabolic syndrome and its influencing factors among the Chinese adults: The China Health and Nutrition Survey in 2009. Prev Med.

[35] Kim MH, Kim MK, Choi BY, Shin YJ (2004). Prevalence of the metabolic syndrome and its association with cardiovascular diseases in Korea. J Korean Med Sci.

[36] Malik S, Wong ND, Franklin SS, Kamath TV, L'Italien GJ, Pio JR (2004). Impact of the metabolic syndrome on mortality from coronary heart disease, cardiovascular disease, and all causes in United States adults. Circulation.

[37] Zhang YH, Dai HQ, Wu JJ, Liu Z, Zhang L (2014). Investigation on prevalence of the major chronic diseases and its related risk factors among adult residents in Daxing district of Beijing city. Chin J Prey ContrChron Dis.

[38] Cao YL, Lei M, Liu DB, Wu JH, Xia XK (2015). Investigation on prevalence of metabolic syndrome and risk factors among adults in Changde. Lab Med Clin.

[39] Chen QY, Luo ZJ, Xia N, Lu H, Qin WW, Peng YH (2007). The prevalence of metabolic syndrome in Hart and Zhuang Chinese in Guangxi. Chin J EndocrinolMetab.

[40] Li H, Shi LX, Zhang Q, Peng NC, Xu SJ, Zhuang HJ (2013). Prevalence of metabolic syndrome in Chinese population aged over 40 in Guiyang. Chin J EndocrinolMetab.

[41] Fu SY, Zhao YJ, Wu S, Zhao JB, Dong LH, Wang FM (2010). Epidemiological characteristics of metabolic syndrome and its correlation factors in Harbin. Chin J EndocrinolMetab.

[42] Tao R, Wu M, Qin Y, Su J, Zhang YQ, Lv SR (2015). Epidemiological characteristics of metabolic syndrome and comparison between its different diagnostic criteria in adults of Jiangsu Province. J Jilin Univ.

[43] Xu DM, Feng CY, Sun LH (2010). Prevalence of rural women with metabolic syndrome. Prev Med.

[44] Lu W, Liu MX, Li R, Fu H, Jin TY, Zhang SN (2006). Epidemiological feature of metabolic syndrome in Shangh residents aged 15–74 years. Chin J Prev Med.

[45] Hu Y, Chen SJ, Wei M, Chen JP, Li X, Zhang SW (2008). The epidemiological study of geriatric metabolic syndrome in Shenyang. China Geriatric Care.

[46] Wang WC, Zhang J, Liu SB, Yan SJ, Liang Q, Ren QH (2014). Analysis of HbAlc Profile and Glucose Tolerance State in Middleaged and senior people with metabolic syndrome in Shijiazhuang City. Modern Prevent Med.

[47] Du YH, Liu L, Zhang YP, Zeng ZP, Wei ZL, Cheng SGR (2007). Epidemic characteristics of metabolic syndrome and its risk factors among inhabitants in the City of Taiyuan. Dis Surv.

[48] Yu H, Wu QN, Li D, Xu YX, Tian SJ, Dong J (2012). Epidemiologiical Features of Metabolic Syndrome and risk factors in 35–60 years Residents in Suburban Areas of Tianjin. Chinese J Pract Intern Med.

[49] Yu L, Zhang YH, Liu YY, Wu BT, Zhang XY, Tong WJ (2009). Comparison of three diagnosis criteria for metabolic syndrome in Mongolian people of agricultural and pastoral regions. J Endocrinol Invest.

[50] Zhang SQ, Chen P, Wu LP, Yang PL (2007). Epidemiological study on the prevalence of metabolic syndrome among residents older than 50 years in Wenzhou. Chin J PrevContrChron Non-comnlun Dis.

[51] Zhao FC, Ge JP, Zhao LM, Tian XQ (2009). Epidemiological study on the prevalence of metabolic syndrome among 3293 Residents Aged 20–74 years in Xinjiang. Xinjiang Medicine.

[52] Li SJ, Cao YS, Sun S, Feng W, Wang XM (2012). Epidemiological features of metabolic syndrome among adults in Fenghua. Chin Prev Med.

[53] Deng M, Deng HC, Wang X, Qu H, Chen C, Liu F (2014). The prevalence of metabolic syndrome in residents aged over 35 years in Chongqing. Chin J EndocrinolMetab.

[54] Ye QY, Xiang XQ, Ni XM, Qiu JH, Xu WW, Ye Z (2012). A Cross-sectional study of metabolic syndrome in population aged over 18. Zhejiang Prevent Med.

[55] Ta JGL, Yao XG, Zhang DL, Wang HM, Zhou L, Hong J (2013). The prevalence of metabolic syndrome in Xinjiang in 2010. Chin Prev Med.

[56] Li CH, Guo SX, Ma RL, Ding YS, Guo H, Liu JM (2012). The epidemic situation of metabolic syndrome among the Uygur in Kashgar of Xinjiang in 2010. Chin Prev Med.

[57] Li YQ, Chen Y, Liu X, Liang Y, Shao X, Zhang Y (2014). Metabolic syndrome and chronic kidney disease in a Southern Chinese population. Nephrology (Carlton).

[58] Sun M, Cao M, Fu Q, Zhu Z, Meng C, Mao J (2014). Association of calcaneal quantitative ultrasound parameters with metabolic syndrome in middle-aged and elderly Chinese: a large population-based cross-sectional study. BMC EndocrDisord.

[59] Yu M, Xu CX, Zhu HH, Hu RY, Zhang J, Wang H (2014). Associations of cigarette smoking and alcohol consumption with metabolic syndrome in a male Chinese population: a cross-sectional study. J Epidemiol.

[60] He Y, Jiang B, Wang J, Feng K, Chang Q, Li F (2006). Prevalence of the metabolic syndrome and its relation to cardiovascular disease in an elderly Chinese population. J Am CollCardiol.

[61] Zhou HC, Lai YX, Shan ZY, Jia WP, Yang WY, Lu JM (2014). Effectiveness of different waist circumference cut-off values in predicting metabolic syndrome prevalence and risk factors in adults in China. Biomed Environ Sci.

[62] Peng X, Li Y, Li J, Liu FY, Peng YM, Sun L (2009). Metabolic syndrome and chronic kidney disease in a rural adult population of Hunan province, China. Chin J Epidemiol.

[63] Tan XU, Zhang YH, Liang YU, Tong WJ (2009). Prevalence of metabolic syndrome and its risk factors in inner Mongolia, China. Acta Csrdiol.

[64] Li G, de Courten M, Jiao S, Wang Y (2010). Prevalence and characteristics of the metabolic syndrome among adults in Beijing, China. Asia Pac J ClinNutr.

[65] Zhao YL, Yan H, Yang R, Li Q, Dang S, Wang YY (2014). Prevalence and determinants of metabolic syndrome among adults in a rural area of Northwest China. PLoS One.

[66] Xu F, Zhang HF, Zhu ZY, Yao WM, Li J, Guo J (2011). Prevalence and risk factors of metabolic syndrome among 18 to 74 years old rural population of Gaoyou in Jiangsu. Chin J Hyperten.

